# Screening and clinical significance of lymph node metastasis‐related genes within esophagogastric junction adenocarcinoma

**DOI:** 10.1002/cam4.4065

**Published:** 2021-06-21

**Authors:** Rui Han, Gang Chen, Meng Li, Zhong‐Min Peng, Lin Xu

**Affiliations:** ^1^ Department of Thoracic Surgery Shandong Provincial Hospital Affiliated to Shandong First Medical University Shandong Province P. R. China

**Keywords:** *CDK6*, esophagogastric junction adenocarcinoma, *LRP1B*, lymph node metastasis, targeted next‐generation sequencing

## Abstract

**Background:**

Despite recent improvements in treatment technologies, such as surgical resection and chemoradiotherapy, the prognosis of patients with esophagogastric junction adenocarcinoma (EJA) remains poor due to early lymph node metastasis. Since few studies have investigated genes associated with lymph node metastasis in EJA, we aimed to screen lymph node metastasis‐associated genes and clarify their expression status and prognostic significance in EJA.

**Methods:**

The differential frequency of mutations between carcinoma and para‐carcinoma tissues from 199 cases with EJA was detected using targeted next‐generation sequencing (tNGS). Following a stratified analysis to determine that gender has no effect on the frequency of gene mutations, lymph node metastasis‐related genes, including *CDK6*, *MET*, *NOTCH1*, and *LRP1B*, were screened, and *CDK6* and *LRP1B* were selected for further study as they displayed significant differences in mutation rates. Differences in their expression status were verified using immunohistochemical (IHC) staining in 18 *CDK6*‐ and 17 *LRP1B*‐mutated samples and a randomly matched control group.

**Results:**

tNGS revealed that *CDK6* and *LRP1B* mutation frequencies were significantly different between EJA cases with (*N* ≥ 1) or without (*N* = 0) lymph node metastasis. In particular, *CDK6* mutation frequency was expected less, whereas that of *LRP1B* was remarkably higher in cases with stage N0 than in those with stage *N* ≥ 1. IHC staining confirmed significant differences in *CDK6* and *LRP1B* expression status between the study and control cohorts. Chi‐square tests revealed that a high *CDK6* expression status correlated significantly with smoking history (*p* = 0.044), T stage (*p* = 0.035), N stage (*p* = 0.000), and advanced TNM stage (*p* = 0.001) in EJA, whereas a high *LRP1B* expression status only correlated with BMI (*p* = 0.013) and N stage (*p* = 0.000). Furthermore, as confirmed by survival status investigation, a high *LRP1B* expression status predicted good prognosis, and a high *CDK6* expression status was an independent predictor of poor prognosis in patients with EJA.

**Conclusions:**

Taken together, the findings of this study demonstrate that a high *CDK6* and *LRP1B* expression status promotes and inhibits lymph node metastasis in patients with EJA, respectively, suggesting that both *CDK6* and *LRP1B* are significantly potential predictors of lymph node metastasis and prognosis in EJA.

## Graphical abstract

Screening of lymph node metastasis‐related genes by targeted next‐generation sequencing in of esophagogastric junction adenocarcinoma. CDK6 and LRP1B were selected for further study to clarify their expression and prognostic significance in AEG.

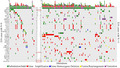

## 1 INTRODUCTION

In 2018, stomach carcinoma (gastric carcinoma with cardia and non‐cardia features combined) became the fifth most prevalent carcinoma (5.7%) and the third‐leading cause of carcinoma‐related death (8.2%) globally, accounting for 782,685 deaths and 1,033,701 new cases.^1^ Though the incidence of distal gastric carcinoma has gradually declined in recent decades in regions, such as North America, Europe, and Asia, the incidence of esophagogastric junction adenocarcinoma (EJA) has increased from 22.3% (1988–1992) to 35.7% (2008–2012; *p* < 0.001).^2^ Despite improvements in comprehensive treatment technologies, such as surgical resection, chemotherapy, and radiotherapy, patients with EJA have a five‐year surviving rate below 30%.^3^ Due to the associated increasing relative frequency of occurrence and mortality, EJA remains one of the most severe socioeconomic burdens; therefore, it is essential to identify novel therapeutic targets for EJA.

Lymph node metastasis occurs in both the chest and abdomen and is the primary type of metastasis in EJA. In addition, the lymph node metastasis rate is particularly high in patients with EJA. For instance, Yoshikawa et al^4^ reported a lymph node metastasis rate of 64.3% in patients with Siewert type II EJA, whereas Goto et al^5^ reported rates of 42.9% and 74%, respectively, in stage T1 and stages T2‐4 Siewert type II and type III EJA. Thus, the molecular mechanisms underlying lymph node metastasis in EJA should be elucidated to identify novel therapeutic targets.

Over the past few years, the rapid advancement of gene chips and third‐generation sequencing technologies has opened up new avenues for studying the molecular mechanisms underlying the biological behavior of malignant tumors. Indeed, genome sequencing has revealed unique genomic characteristics, made up for the lack of histopathological data, and led to the identification of crucial biomarkers and potential novel molecular targets to improve the precise clinical treatment and prognosis of carcinoma. The present study determined the differential expression status of mutant genes in carcinoma and para‐carcinoma tissues from 199 patients with EJA using next‐generation sequencing (NGS) and preliminarily screened lymph node metastasis‐associated genes, including *CDK6*, *MET*, and *NOTCH1*, as well as low‐density lipoprotein receptor‐related protein 1B (*LRP1B*). After verifying gene expression status using immunohistochemical (IHC) staining, we performed correlation analyses on *CDK6* and *LRP1B* based on survival data and clinicopathological characteristics. Taken together, our findings suggest that a high *CDK6* expression status is an independent prognosis‐related factor in patients with EJA.

## 2 MATERIALS AND METHODS

### 2.1 Cases and specimens

Tissue specimens were obtained from 199 patients with EJA who underwent curative resection with lymph node dissection at the Thoracic and Gastrointestinal Surgery Department of the Shandong Provincial Hospital Affiliated to the Shandong First Medical University from January 2012 to November 2018. Among them, the number of EJA patients with (*N* ≥ 1) and without (*N* = 0) lymph node metastasis was 152. The patients included 169 males and 30 females who were selected according to the following criteria: (1) did not receive preoperative neoadjuvant chemotherapy; (2) did not have synchronous tumors in other organs and tissues; and (3) did not have multiple metachronous tumors in the esophagogastric junction. The tumor stage was determined based on the American Joint Committee on Carcinoma (AJCC) eighth edition carcinoma staging manual. The carcinoma tissues were collected from the center of the lesions, whereas the para‐carcinoma tissues were collected from the contralateral normal tissue at the esophagogastric junction 2 cm away from the lesion. It should be noted that care must be taken when collecting samples to ensure that the para‐carcinoma tissue is normal tissue with no contamination of tumor tissue. Patients provided prior approval and written informed consent regarding the applications of their tumor tissues and clinical records. We performed this study rigorously, complying with the Declaration of Helsinki, and obtained approval from the Medical Ethical Committee of the Shandong Provincial Hospital Affiliated with Shandong First Medical University.

### 2.2 Targeted NGS (tNGS) and genetic analysis

All formalin‐fixed and paraffin‐embedded (FFPE) tumor tissues were validated by pathologists to ensure that the tumor cell percentage in the respective specimen was >20%. The samples were then transferred to the American Pathologists College (CAP)/Clinical Laboratory Improvement Amendments (CLIA) certified laboratory in OrigiMed (Shanghai, China). A total of 50–250 ng DNA was extracted from the tissue sections that were not stained to analyze genetic alterations using the NGS‐based Yuansu^TM^450 gene panel (OrigiMed, Shanghai, China), which covered all coding exons of 450+ carcinoma‐associated DNA segments and 64 introns of 39 DNA segments systematically arranged in solid tumors. The resulting libraries were diluted to 1.05 nM and subsequently processed under an average depth of 800× using the Illumina NextSeq‐500 platform (Illumina), as described previously.^6^ Genomic alterations, including gene fusions, gene rearrangements, copy number variations, short and long insertions/deletions (indels), and single nucleotide changes, were identified according to previously described methods^7^ and subjected to advanced analysis.^8^

Due to the large gap in the number of male and female cases, we first conducted a stratification analysis of the 199 patients according to gender to eliminate the influence of gender on gene mutation frequency. After the preliminary screening of lymph node metastasis‐related genes, including *CDK6*, *MET*, *NOTCH1*, and *LRP1B*, 18 cases with *CDK6* mutation and 17 cases with *LRP1B* mutation were screened out, with the remaining cases screened as the gender‐ and age‐matched control group for subsequent IHC staining. The clinicopathological characteristics of patients in the *CDK6* and *LRP1B* groups, including TNM stage, lymph node spread, tumor diameter, differentiation extent, age, and gender, are shown in Table 1.

**TABLE 1:** Relationship between *CDK6* and *LRP1B* expression status in esophagogastric junction adenocarcinoma tissues and clinicopathological characteristics

Clinico‐pathological variable	*n* = 36	*CDK6* expression status (%)		*p*‐value	*n* = 34	*LRP1B* expression status (%)		*p*‐value
		Strongly/moderately positive (*n* = 18)	Negative/weakly positive (*n* = 18)			Strongly/moderately positive (*n* = 17)	Negative/weakly positive (*n* = 17)	
Age (years)								
<60	14 (38.9)	7	7	1.0	14 (41.2)	7	7	1.0
≥60	22 (61.1)	11	11		20 (58.8)	10	10	
Gender								
Male	24 (66.7)	12	12	1.0	32 (94.1)	16	16	1.0
Female	12 (33.3)	6	6		2 (5.9)	1	1	
Smoking history								
No	20 (55.6)	7	13	**0.044**	18 (52.9)	7	11	0.169
Yes	16 (44.4)	11	5		16 (47.1)	10	6	
BMI								
Underweight	1 (2.8)	1	0	0.189	0 (0)	0	0	**0.013**
Normal	21 (58.3)	8	13		13 (38.2)	3	10	
Overweight	14 (38.9)	9	5		21 (61.8)	14	7	
Tumor size (cm)								
<5	16 (44.4)	8	8	0.877	17 (50)	9	8	0.732
≥5	19 (55.6)	10	9		17 (50)	8	9	
Differentiation								
Poor	23 (63.9)	13	10	0.105	24 (70.6)	14	10	0.132
Moderate	9 (25)	5	4		10 (29.4)	3	7	
High	4 (11.1)	0	4		0 (0)	0	0	
Tumor location								
Proximal	27 (75)	14	13	0.7	24 (70.6)	14	10	0.132
Distal	9 (25)	4	5		10 (29.4)	3	7	
Siewert type								
Siewert I	4 (11.1)	2	2	0.926	3 (8.8)	1	2	0.21
Siewert II	23 (63.9)	12	11		21 (61.8)	13	8	
Siewert III	9 (25)	4	5		10 (29.4)	3	7	
TNM classification								
T stage								
T1	0 (0)	0	0	**0.035**	1 (2.9)	0	1	0.335
T2	3 (8.3)	0	3		5 (14.7)	3	2	
T3	26 (72.2)	12	14		23 (67.6)	10	13	
T4	7 (19.5)	6	1		5 (14.7)	4	1	
N stage								
N0	4 (11.1)	0	4	**0.000**	14 (41.2)	14	0	**0.000**
N1	9 (25)	1	8		6 (17.6)	2	4	
N2	8 (22.2)	2	6		5 (14.7)	1	4	
N3	15 (41.7)	15	0		9 (26.5)	0	9	
TNM stage								
Ⅰ	0 (0)	0	0	**0.001**	4 (11.8)	3	1	0.055
Ⅱ	11 (30.6)	1	10		13 (38.2)	9	4	
Ⅲ	25 (69.4)	17	8		17 (50)	5	12	
Ⅳ	0 (0)	0	0		0 (0)	0	0	

Bold indicates significance values *p* < 0.05

### 2.3 IHC staining

To confirm differential *CDK6* and *LRP1B* expressions between the study and control groups, the tumor tissues screened and matched by tNGS were subjected to IHC staining according to the manufacturer's instructions (Abcam). FFPE EJA tumor tissues from the *CDK6* (*n* = 36) and *LRP1B* (*n* = 34) groups were sectioned (5‐μm thick) and subsequently deparaffinized with xylene and rehydrated using an ethanol gradient. After blocking endogenous peroxidase for 15 min using 3% hydrogen peroxide in methanol, antigen retrieval was carried out using sodium citrate buffer (pH 6.0), followed by the incubation of the sections in 5% goat serum for 20 min prior to IHC staining. Next, the sections were incubated with rabbit anti‐human *CDK6*/*LRP1B* (1:200 dilution, G1213; Abcam) primary antibodies for 16 h at 4℃, and then with horseradish peroxidase binding anti‐rabbit secondary antibodies (1:200 dilution, G1213; Abcam) for 30 min at 37℃. After staining with diaminobenzidine and counterstaining with hematoxylin for 2 min, the sections were mounted with neutral resin and imaged using an Olympus BX41 microscope (Ina‐shi)/Logene LG1000 (Wuxi) Digital Camera System.

### 2.4 IHC analysis

Immunohistochemical staining for *CDK6* and *LRP1B* was defined as positive when buffy staining was observed inside the cell membrane and/or cytoplasm. The results were evaluated by two different senior pathologists who have specialized in EJA and were blinded to clinical data, such as pathological type and clinical phase. Five different fields of view were observed randomly per section under high magnification (40 × 10). The IHC staining results were semi‐quantitatively classified and scored into the following five groups according to the average percentage of stained cells: 0 (no stained cells), 1 (1%–25%), 2 (26%–50%), 3 (51%–75%), and 4 (76%–100%). Based on the presence of brownish‐yellow color in the cell membrane and/or cytoplasm, the staining intensity was scored as follows: 0 (no staining), 1 (pale‐yellow), 2 (brownish‐yellow), and 3 (dark‐brown). The total score was obtained as the product of the percentage and intensity scores for each section: 0, negative (−); 1–3, weakly positive (+); 4–6, positive (++); 8–12, and strongly positive (+++). The samples were divided into two cohorts based on the overall score: 0–3, negative and weakly positive; 4–12, moderately and significantly positive.

### 2.5 Statistical analysis

**FIGURE 1:**
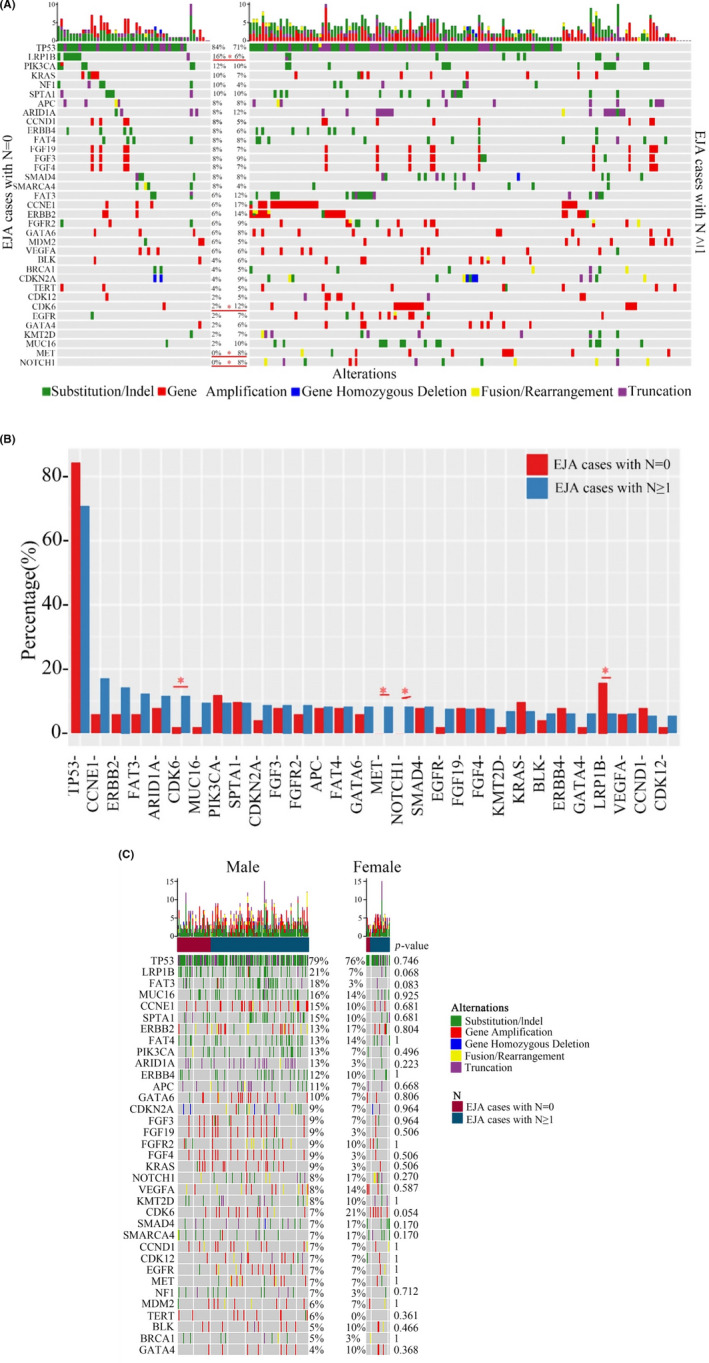
(A) Targeted next‐generation sequencing (tNGS) and genetic analysis of carcinoma and carcinoma‐adjacent tissues from stage 199 patients with esophagogastric junction adenocarcinoma (EJA) at stage N0 or *N* ≥ 1. (B) Mutation frequencies of *CDK6*, *MET*, *NOTCH1*, and *LRP1B* differed significantly between patients with stages N0 and *N* ≥ 1. (C) The mutation frequencies of *CDK6*, *LRP1B*, and other genes in 163 male and 30 female patients

Statistical analysis was performed using SPSS v26.0 (IBM). The correlation between *CDK6*/*LRP1B* expression status and the corresponding clinicopathological characteristics of each patient was assessed by chi‐square or Fisher's exact tests (Table 1). Further, chi‐square test was used to analyze the differences in the mutation frequencies of different genes in different genders (Figure 1C). Overall survival (OS) curves were plotted using the Kaplan–Meier approach, and statistics‐related differences in survival rates were examined using the log‐rank test. Univariate Cox analysis was used to assess the correlation of *CDK6*/*LRP1B* expression status and clinicopathological variables with patient survival. Single prognosis‐related predictors for EJA were identified using multiple‐variate Cox proportional hazards regression analysis. Differences with a *p*‐value < 0.05 were considered significant.

## 3 RESULTS

### 3.1 CDK6 and LRP1B mutation frequencies are related to lymph node metastasis in EJA

The present study detected the mutated genes in EJA and EJA‐adjacent tissues from 199 patients with EJA using tNGS. *CDK6*, *MET*, *NOTCH1*, and *LRP1B* mutation frequencies showed significant differences in cases with (*N* ≥ 1) or without (*N* = 0) lymph node metastasis (Figure 1B). Specifically, the mutation frequencies of *CDK6*, *MET*, and *NOTCH1* noticeably lower in cases with stage N0 than in those with stage *N* ≥ 1, whereas *LRP1B* mutation frequency was remarkably higher in cases without lymph node metastasis. Though no *MET* and *NOTCH1* mutations were observed in cases with stage N0, an 8% mutation rate was noted in cases with stage *N* ≥ 1. Moreover, the mutation rate of *CDK6* was 2% in cases with stage N0 and 12% in cases with stage *N* ≥ 1, whereas the mutation rate of *LRP1B* was 16% in cases with stage N0 and 6% in cases with stage *N* ≥ 1 (Figure 1A). Stratification analysis based on gender showed that although the mutation frequencies of different genes in cases of different genders were different, the difference was not statistically significant (Figure 1C). Thus, these results suggest that high *CDK6* and low *LRP1B* mutation rates have a close association with lymph node metastasis in patients with EJA.

### 3.2 CDK6 and LRP1B expression status is related to the clinicopathological features of patients with EJA

**FIGURE 2:**
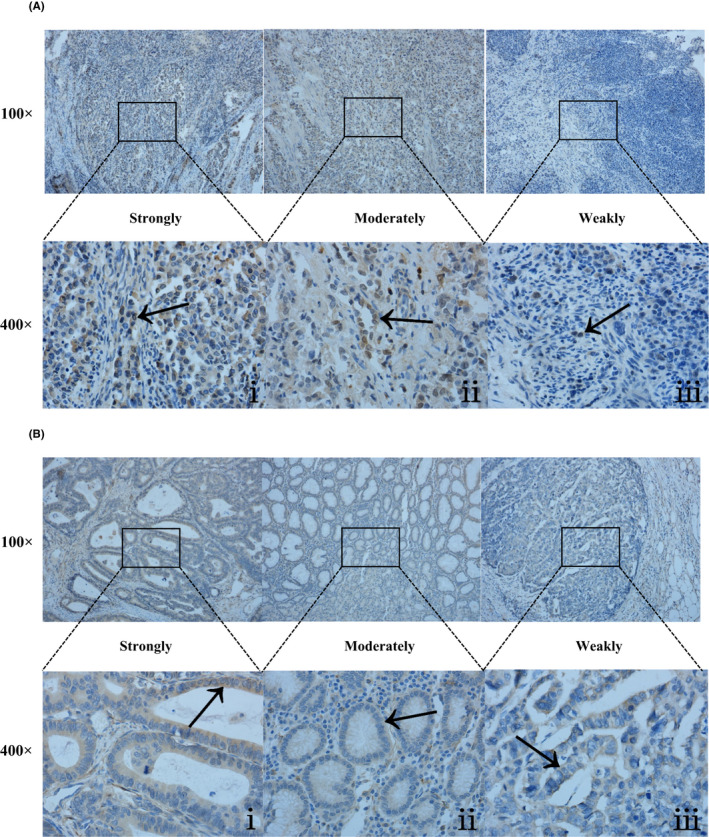
Representative photomicrographs of *CDK6* and *LRP1B* immunohistochemical staining in esophagogastric junction adenocarcinoma (EJA) tissues. (A) Strongly positive expression status (ⅰ), moderately positive expression status (ⅱ), and weakly positive expression status (ⅲ) of *CDK6* in EJA tissues. (B) Strongly positive expression status (ⅰ), moderately positive expression status (ⅱ), and weakly positive expression status (ⅲ) of *LRP1B* in EJA tissues

To verify the differential expression status between *CDK6* and *LRP1B* in patients with (study group) or without (control group) mutations, we performed IHC staining on tumor tissues from 36 and 34 patients, who were screened and matched using tNGS, from the *CDK6* and *LRP1B* groups, respectively. The *CDK6* group included 12 females and 24 males aged 62.1 years on average at diagnosis, while the *LRP1B* group included 2 females and 32 males with a mean age of 60.03 years at diagnosis. We found that *CDK6* and *LRP1B* expression status differed significantly between the study and control groups. In particular, *CDK6* was mainly expressed in the nucleus of EJA tissues, whereas *LRP1B* was mainly expressed in the cytoplasm, as illustrated by the representative IHC photomicrographs shown in Figure 2. Additionally, we examined the association between *CDK6* and *LRP1B* expression status and the clinicopathological characteristics of patients with EJA (Table 1). Chi‐square tests revealed that a high *CDK6* expression status significantly correlated with smoking history (*p* = 0.044), T stage (*p* = 0.035), N stage (*p* = 0.000), and advanced TNM stage (*p* = 0.001) in EJA, whereas a high *LRP1B* expression status only correlated with BMI (*p* = 0.013) and N stage (*p* = 0.000; Table 1). Therefore, these results indicate that the expression status of both *LRP1B* and *CDK6* is closely related to lymph node metastasis in patients with EJA. A high *CDK6* expression status may promote lymph node metastasis, while a high *LRP1B* expression status may inhibit lymph node metastasis.

### 3.3 High CDK6 expression status in EJA tissues is an independent predictor of poor prognosis

**FIGURE 3:**
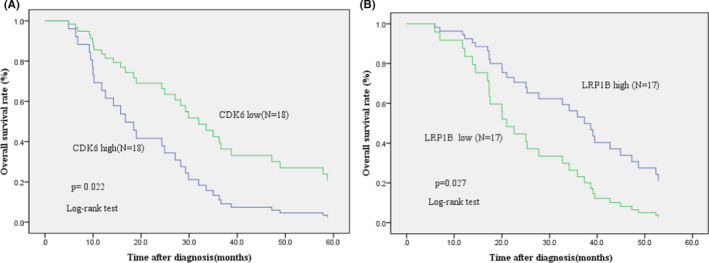
A high *CDK6* expression status and low *LRP1B* expression status are prognostic predictors in patients with esophagogastric junction adenocarcinoma (EJA). (A) Patients with EJA and a high *CDK6* expression status had a significantly poor prognosis (overall survival [OS]) than those with a low expression status (*p* = 0.022, log‐rank test). (B) Patients with EJA and a low *LRP1B* expression status had a significantly favorable prognosis (OS) than those with a high expression status (*p* = 0.027, log‐rank test)

To investigate whether *CDK6* expression status is associated with the survival of patients with EJA, we obtained follow‐up information for 36 patients with EJA from the *CDK6* group. Kaplan–Meier analysis indicated that cases with a high *CDK6* expression status had a shorter OS than those with a low *CDK6* expression status (Figure 3A, log‐rank test *p* = 0.022). Moreover, univariate Cox regression analysis revealed that T stage (hazard ratio (HR) = 3.258, 95% confidence interval (CI) = 1.377–7.71, *p* = 0.007), tumor location (HR = 0.367, 95% CI = 0.148–0.906, *p* = 0.03), and a high *CDK6* expression status (HR = 2.358, 95% CI = 1.132–4.91, *p* = 0.022) were prognosis‐related risk factors for OS in the *CDK6* group (Table 2). After eliminating the influence of other factors using multivariate Cox proportional hazards regression analysis, we confirmed that tumor location (HR = 0.037, 95% CI = 0.006–0.232, *p* = 0.000) and a high *CDK6* expression status (HR = 17.815, 95% CI = 1.706–186.035, *p* = 0.016) were independent risk factors for OS in the *CDK6* group (Table 2). Taken together, these results revealed that a high CDK6 expression status is an independent prognosis‐related factor in EJA.

**TABLE 2:** Univariate and multivariate Cox regression analyses of risk factors in the *CDK6* group of 36 patients with EJA

Parameter	OS (univariate)			OS (multivariate)		
	HR	95% CI	*p*‐value	HR	95% CI	*p*‐value
Gender (male vs. female)	0.816	0.384–1.737	0.598	0.304	0.080–1.156	0.081
Age (<60 years vs. ≥60 years)	0.907	0.439–1.876	0.793	2.985	0.738–12.067	0.125
BMI	1.238	0.608–2.523	0.557	1.680	0.726–3.889	0.226
Tumor size (<5 cm vs. ≥5 cm)	1.4	0.675–2.902	0.366	4.089	1.284–13.019	**0.017**
Tumor location (proximal vs. distal)	0.367	0.148–0.906	**0.03**	0.037	0.006–0.232	**0.000**
T stage	3.258	1.377–7.71	**0.007**	2.136	0.638–7.157	0.219
N stage	1.326	0.935–1.88	0.114	0.848	0.314–2.286	0.744
TNM stage	1.607	0.75–3.442	0.223	3.673	0.798–16.905	0.095
NLR	0.997	0.907–1.096	0.956	1.367	1.106–1.689	**0.004**
PLR	0.999	0.995–1.003	0.574	1.002	0.995–1.009	0.560
*CDK6* expressing state (high vs. low)	2.358	1.132–4.91	**0.022**	17.815	1.706–186.035	**0.016**

Abbreviations: CI, confidence interval; EJA, Esophagogastric junction adenocarcinoma; HR, hazard ratio; NLR, neutrophil‐to‐lymphocyte rate; OS, overall survival; PLR, platelet‐to‐lymphocyte rate.

Bold indicates significance values *p* < 0.05

### 3.4 High LRP1B expression status in EJA tissues predicts a favorable prognosis

Next, we examined the follow‐up data of 34 patients in the *LRP1B* group, and the Kaplan–Meier analysis indicated that a high *LRP1B* expression status is related to a longer OS in patients with EJA (Figure 3B, log‐rank test *p* = 0.027). In addition, as revealed by univariate Cox regression analysis, N stage (HR = 1.566, 95% CI = 1.124–2.182, *p* = 0.008), tumor location (HR = 2.762, 95% CI = 1.205–6.331, *p* = 0.016), platelet‐to‐lymphocyte ratio (PLR; HR = 1.011, 95% CI = 1.004–1.019, *p* = 0.016), and a low *LRP1B* expression status (HR = 0.432, 95% CI = 0.206–0.909, *p* = 0.027) were prognosis‐related risk factors for OS in the *LRP1B* group (Table 3). Multiple‐variate Cox proportional hazards regression analysis further revealed that N stage (HR = 3.151, 95% CI = 1.307–7.594, *p* = 0.011) was an independent risk factor for OS in the *LRP1B* group (Table 3), indicating that a low *LRP1B* expression status promotes lymph node metastasis in patients with EJA.

**TABLE 3:** Univariate and multivariate Cox regression analyses of risk factors in the *LRP1B* group of 34 patients with EJA

Parameter	OS (univariate)			OS (multivariate)		
	HR	95% CI	*p*‐value	HR	95% CI	*p*‐value
Gender (male vs. female)	1.412	0.328–6.074	0.643	1.703	0.268–10.806	0.572
Age (<60 years vs. ≥60 years)	1.207	0.58–2.512	0.614	1.246	0.508–3.058	0.631
BMI	1.023	0.485–2.158	0.953	2.636	0.872–7.976	0.086
Tumor size (<5 cm vs. ≥5 cm)	0.95	0.464–1.947	0.889	0.713	0.277–1.836	0.483
Tumor location (proximal vs. distal)	2.762	1.205–6.331	**0.016**	2.560	0.754–8.691	0.132
T stage	1.136	0.673–1.916	0.633	1.795	0.470–6.852	0.392
N stage	1.566	1.124–2.182	**0.008**	3.151	1.307–7.594	**0.011**
TNM stage	1.396	0.82–2.375	0.219	0.402	0.094–1.726	0.220
NLR	1.287	0.736–2.249	0.376	1.872	0.827–4.238	0.133
PLR	1.011	1.004–1.019	**0.016**	0.995	0.981–1.009	0.490
*LRP1B* expressing state (high vs. low)	0.432	0.206–0.909	**0.027**	0.926	0.187–4.589	0.925

Abbreviations: CI, confidence interval; EJA, Esophagogastric junction adenocarcinoma; HR, hazard ratio; NLR, neutrophil‐to‐lymphocyte rate; OS, overall survival; PLR, platelet‐to‐lymphocyte rate.

Bold indicates significance values *p* < 0.05

## 4 DISCUSSION

The relative frequency of occurrence of gastric carcinoma and the related mortality have declined worldwide in recent decades,^9^ mainly due to a gradual decline in the prevalence of *Helicobacter pylori* infection and tobacco smoking, as well as improved dietary habits; however, the burden of gastric carcinoma remains high in several countries in Asia, Eastern Europe, and South America.^1^ In addition, the incidence of EJA has substantially increased during the same period, and its prognosis remains poor, despite the significant improvements in treatment strategies, such as surgical resection and chemoradiotherapy, mainly due to early lymph node metastasis and two‐field lymph node metastasis.^10^ Accordingly, the molecular mechanisms of lymph node metastasis in EJA should be elucidated for providing new therapeutic targets. In this study, tNGS revealed that the frequencies of *CDK6*, *MET*, and *NOTCH1* mutations were noticeably higher in EJA tissues with lymph node metastasis than in adjacent tissues, while the frequency of *LRP1B* mutations was significantly lower in cases with lymph node metastasis than in para‐cancerous tissues. Moreover, IHC staining confirmed that *CDK6* expression was remarkably higher, whereas that of *LRP1B* was lower in EJA tissues than in adjacent tissues. Finally, multivariate and univariate Cox analyses using clinicopathological parameters confirmed a high *CDK6* expression status as an independent risk factor for OS in EJA.

Exons are the coding regions of eukaryotic genes that undergo transcription, and they are translated into corresponding proteins that perform specific biological functions. Though exons account for less than 1% of the human genome, they contain approximately 85% of all the genetic information in humans. Unlike traditional sequencing techniques, such as Sanger sequencing, NGS simultaneously sequences thousands‐to‐millions of short nucleic acid sequences in a massively parallel way to generate large volumes of sequence data, thus providing a cost‐effective approach for detecting multiple genetic alterations.^11^ Salem et al^12^ used the NextSeq platform to perform NGS of genomic DNA isolated from the tumor tissues of 3342 gastroesophageal carcinomas, including esophageal squamous cell carcinoma, esophageal adenocarcinoma, and gastric adenocarcinoma, and revealed that esophageal squamous cell carcinoma exhibits a molecular profile different from that of esophageal adenocarcinoma and gastric adenocarcinoma. In contrast, the molecular profile of esophageal adenocarcinoma was highly similar to that of gastric adenocarcinoma. Another study involving targeted deep sequencing at 739 hotspots in 46 carcinoma‐related genes from 92 EJA and 75 gastric adenocarcinoma resection specimens revealed that the mutation frequencies of specific carcinoma‐related genes and several clinicopathological characteristics differed between EJA and gastric adenocarcinoma.^13^ Consistently, in this study, tNGS revealed that the mutation frequencies of *CDK6* and *LRP1B* significantly differed between EJA patients with stages N0 and *N* ≥ 1, suggesting that a high *CDK6* expression status and a low *LRP1B* expression status may be related to lymph node metastasis in patients with EJA. Together, the findings of this study provide new avenues for researching the molecular mechanisms underlying lymph node metastasis in EJA and experimental and genomic data for subsequent studies.

*CDK6*, encoded by the human *PLSTIRE* gene,^14^ is located on chromosome 7q21‐22,^15^ contains 7 exons, and spans 226 kb of genomic DNA. Previous studies have shown that *CDK6* can activate the CDK‐cyclinD‐Rb‐E2F pathway, enabling the transcription factor E2F to activate tumor gene transcription and force the cell cycle to enter the S phase, thereby promoting the growth, migration, and metastasis of various malignant tumors, including pancreatic neuroendocrine,^16^ cervical,^17^ bladder,^18^ gastric,^19^ epithelial ovarian,^20^ and breast carcinomas.^21^ Recent studies have also revealed that *CDK6* is regulated by upstream noncoding RNA, and it indirectly affects tumor lymph node metastasis and other biological behaviors. For instance, Xue et al^22^ reported that binding to hsa_circ_0081143 and miR646 effectively reversed the inhibition of *CDK6*, which was closely related to lymph node metastasis and was a poor prognosis predictor in patients with gastric carcinoma. Similarly, Pan et al^23^ discovered that miR‐4429 inhibits cell proliferation, migration, invasion, and epithelial‐mesenchymal transition (EMT) by targeting *CDK6* in clear cell renal cell carcinoma and its expression status is closely related to lymph node metastasis. Together, these studies have validated the potential of *CDK6* as an antitumor therapeutic target, making the study of CDK4/6 inhibitors to treat malignant tumors an area of active research; indeed, the CDK4/6 inhibitors palbociclib, ribociclib, and abemaciclib have now been approved by the U.S. Food and Drug Administration. Consistently, we found that *CDK6* was overexpressed in EJA tissues with lymph node metastasis and that a high *CDK6* expression status is an independent predictor of poor prognosis in patients with EJA.

*LRP1B* (initially referred to as *LRP*‐*DIT*) is a new member of the low‐density lipoprotein receptor gene family^24^ and was originally identified and named as a candidate tumor suppressor gene by Liu et al when using a probe to detect homozygous deletions on chromosome 2q21.2 in kidney and bladder carcinoma cell lines.^25^ An increasing number of studies have shown that *LRP1B* is underexpressed due to homozygous gene deletions in non‐small cell lung carcinoma,^25^ glioblastoma,^26^ urothelial carcinomas,^27^ and other malignant tumors. Besides, recent studies have suggested that epigenetic silencing due to aberrant promoter methylation or histone deacetylation may be necessary for *LRP1B* inactivation in thyroid carcinoma,^28^ gastric carcinoma,^29^ and renal cell carcinoma.^30^ Additionally, according to Wang et al,^31^ *LRP1B* downregulation promotes the expression of the EMT markers N‐cadherin and Snail, suggesting that it may promote the migration of colon carcinoma cells by inducing EMT. Moreover, the application of 5‐Aza‐DCYD methylation inhibitors to upregulate *LRP1B* expression has been found to partially restore the inhibition of tumor proliferation and migration in multiple malignant tumors, such as oral squamous cell carcinoma,^32^ esophageal squamous cell carcinoma,^33^ gastric carcinoma,^29^ and kidney carcinoma.^30^ In this study, we found that *LRP1B* was underexpressed in EJA tissues with lymph node metastasis and that a high *LRP1B* expression status may inhibit lymph node metastasis and predict a favorable prognosis.

In recent years, numerous studies have reported that the inexpensive and readily available preoperative prognostic biomarkers neutrophil‐to‐lymphocyte ratio (NLR) and platelet‐to‐lymphocyte ratio (PLR) are prominently associated with ineffective prognostic prediction in patients with various malignant tumors, including gastric carcinoma,^34^ colorectal carcinoma,^35^ esophageal squamous cell carcinoma,^36^ non‐small cell lung carcinoma,^37^ cervical carcinoma,^38^ and breast carcinoma.^39^ Furthermore, Kim et al^34^ reported that a high NLR and PLR expression status is significantly associated with ineffective prognostic prediction, which is consistent with our findings that PLR is a prognosis‐related risk factor for OS and predicts poor prognosis in patients with EJA and *LRP1B* mutations.

There are several limitations to this study. First, the results are inevitably biased, as this was a single‐center retrospective trial with small sample size and potentially confounding variables. Second, research on upstream and downstream molecular mechanisms of *CDK6*/*LRP1B* was not comprehensive in this study, and thus multi‐center prospective studies with a larger sample size are needed to confirm the role of *CDK6*/*LRP1B* in the progression and development of EJA.

## 5 CONCLUSIONS

In this study, we demonstrated that a high *CDK6* expression status may promote lymph node metastasis and that a high *LRP1B* expression status inhibits lymph node metastasis in patients with EJA. Moreover, a high *CDK6* expression status was an independent prognosis‐related factor in patients with EJA, while a high *LRP1B* expression status predicted a favorable prognosis. Together, our findings indicate that *CDK6* and *LRP1B* have significant potential as indicators of prognosis and lymph node metastasis in patients with EJA and could be targeted for molecular targeted therapy.

## CONFLICTS OF INTEREST

The authors declare no conflicting interests.

## AUTHOR CONTRIBUTION

Dr. Xu and Dr. Chen contributed to the study design. Dr. Han and Dr. Chen contributed to the drafting of the article. Dr. Han, Dr. Li, and Dr. Xu contributed to data collection. Dr. Chen and Dr. Xu contributed to pathological and immunohistochemistry evaluation. Dr. Xu and Dr. Peng contributed to data bioinformatics analysis. Dr. Xu and Dr. Han participated in data analysis and interpretation and led the article's revision. All authors reviewed and approved the final manuscript.

## ETHICS STATEMENT

The research protocol and consent form were reviewed and approved by the Medical Ethics Committees of Shandong Provincial Hospital Affiliated to Shandong First Medical University (SZRJJ: NO.2020‐003), and written informed consent was signed by each participant.

## Data Availability

The data that support the findings of this study are available from the corresponding author upon reasonable request. The data are not publicly available due to privacy or ethical restrictions.

## References

[1] Bray F , Ferlay J , Soerjomataram I , Siegel RL , Torre LA , Jemal A . Global cancer statistics 2018: GLOBOCAN estimates of incidence and mortality worldwide for 36 cancers in 185 countries. CA Cancer J Clin. 2018;68(6):394‐424. 10.3322/caac.21492 30207593

[2] Liu K , Yang K , Zhang W , et al. Changes of esophagogastric junctional adenocarcinoma and gastroesophageal reflux disease among surgical patients during 1988–2012: a single‐institution, high‐volume experience in China. Ann Surg. 2016;263(1):88‐95. 10.1097/SLA.0000000000001148 25647058 PMC4679348

[3] Siewert JR , Feith M , Stein HJ . Biologic and clinical variations of adenocarcinoma at the esophagogastric junction: Relevance of a topographic‐anatomic subclassification. J Surg Oncol. 2005;90(3):139‐146. 10.1002/jso.20218 15895452

[4] Yoshikawa T , Takeuchi H , Hasegawa S , et al. Theoretical therapeutic impact of lymph node dissection on adenocarcinoma and squamous cell carcinoma of the esophagogastric junction. Gastric Cancer. 2016;19(1):143‐149. 10.1007/s10120-014-0439-y 25414051

[5] Goto H , Tokunaga M , Miki Y , et al. The optimal extent of lymph node dissection for adenocarcinoma of the esophagogastric junction differs between Siewert type II and Siewert type III patients. Gastric Cancer. 2015;18(2):375‐381. 10.1007/s10120-014-0364-0 PMC4371819 24658651

[6] Frampton GM , Fichtenholtz A , Otto GA , et al. Development and validation of a clinical cancer genomic profiling test based on massively parallel DNA sequencing. Nat Biotechnol. 2013;31(11):1023‐1031. 10.1038/nbt.2696 24142049 PMC5710001

[7] Cao J , Chen L , Li H , et al. An accurate and comprehensive clinical sequencing assay for cancer targeted and immunotherapies. Oncologist. 2019;24(12):1294–1302. 10.1634/theoncologist.2019-0236 31409745 PMC6975945

[8] Lin J , Shi J , Guo H , et al. Alterations in DNA damage repair genes in primary liver cancer. Clin Cancer Res. 2019;25(15):4701‐4711. 10.1158/1078-0432.CCR-19-0127 31068370

[9] Sitarz R , Skierucha M , Mielko J , Offerhaus GJA , Maciejewski R , Polkowski WP . Gastric cancer: epidemiology, prevention, classification, and treatment. Cancer Manag Res. 2018;10:239‐248. 10.2147/CMAR.S149619 29445300 PMC5808709

[10] Dresner SM , Lamb PJ , Bennett MK , Hayes N , Griffin SM . The pattern of metastatic lymph node dissemination from adenocarcinoma of the esophagogastric junction. Surgery. 2001;129(1):103‐109. 10.1067/msy.2001.110024 11150040

[11] Metzker ML . Sequencing technologies — the next generation. Nat Rev Genet. 2010;11(1):31‐46. 10.1038/nrg2626 19997069

[12] Salem ME , Puccini A , Xiu J , et al. Comparative molecular analyses of esophageal squamous cell carcinoma, esophageal adenocarcinoma, and gastric adenocarcinoma. The Oncol. 2018;23(11):1319‐1327. 10.1634/theoncologist.2018-0143 PMC6291329 29866946

[13] Li‐Chang HH , Kasaian K , Ng Y , et al. Retrospective review using targeted deep sequencing reveals mutational differences between gastroesophageal junction and gastric carcinomas. BMC Cancer. 2015;15(1):32. 10.1186/s12885-015-1021-7 25656989 PMC4322811

[14] Meyerson M , Harlow E . Identification of G1 kinase activity for cdk6, a novel cyclin D partner. Mol Cell Biol. 1994;14(3):2077‐2086. 10.1128/MCB.14.3.2077 8114739 PMC358568

[15] Thomas JW , Lee‐Lin S‐Q , Green ED . Human‐mouse comparative mapping of the genomic region containing CDK6: localization of an evolutionary breakpoint. Mamm Genome. 1999;10(7):764‐767. 10.1007/s003359901088 10384057

[16] Tang LH , Contractor T , Clausen R , et al. Attenuation of the retinoblastoma pathway in pancreatic neuroendocrine tumors due to increased Cdk4/Cdk6. Clin Cancer Res. 2012;18(17):4612‐4620. 10.1158/1078-0432.CCR-11-3264 22761470

[17] Xiong Y , Li T , Assani G , et al. Ribociclib, a selective cyclin D kinase 4/6 inhibitor, inhibits proliferation and induces apoptosis of human cervical cancer in vitro and in vivo. Biomed Pharmacother. 2019;112:108602. 10.1016/j.biopha.2019.108602 30784916

[18] Liu Z , Wang W , Jiang J , et al. Downregulation of GAS5 promotes bladder cancer cell proliferation, partly by regulating CDK6. PLoS ONE. 2013;8(9):e73991. 10.1371/journal.pone.0073991 24069260 PMC3775789

[19] Feng L , Xie Y , Zhang H , Wu Y . miR‐107 targets cyclin‐dependent kinase 6 expression, induces cell cycle G1 arrest and inhibits invasion in gastric cancer cells. Med Oncol. 2012;29(2):856‐863. 10.1007/s12032-011-9823-1 21264532

[20] Xia B , Yang S , Liu T , Lou G . miR‐211 suppresses epithelial ovarian cancer proliferation and cell‐cycle progression by targeting Cyclin D1 and CDK6. Mol Cancer. 2015;14(1):57. 10.1186/s12943-015-0322-4 25889927 PMC4359570

[21] Cram EJ , Liu BD , Bjeldanes LF , Firestone GL . Indole‐3‐carbinol Inhibits CDK6 Expression in human MCF‐7 breast cancer cells by disrupting Sp1 transcription factor interactions with a composite element in the CDK6 gene promoter. J Biol Chem. 2001;276(25):22332‐22340. 10.1074/jbc.M010539200 11297539

[22] Xue M , Li G , Fang X , Wang L , Jin Y , Zhou Q . hsa_circ_0081143 promotes cisplatin resistance in gastric cancer by targeting miR‐646/CDK6 pathway. Cancer Cell Int. 2019;19(1):25. 10.1186/s12935-019-0737-x 30733646 PMC6359821

[23] Pan H , Hong Y , Yu B , Li L , Zhang X . *miR‐4429* inhibits tumor progression and epithelial‐mesenchymal transition via targeting *CDK6* in clear cell renal cell carcinoma. Cancer Biother Radiopharm. 2019;34(5):334‐341. 10.1089/cbr.2018.2697 30844301

[24] Liu C‐X , Li Y , Obermoeller‐McCormick LM , Schwartz AL , Bu G . The putative tumor suppressor LRP1B, a novel member of the Low Density Lipoprotein (LDL) receptor family, exhibits both overlapping and distinct properties with the LDL receptor‐related protein. J Biol Chem. 2001;276(31):28889‐28896. 10.1074/jbc.M102727200 11384978

[25] Liu C‐X , Musco S , Lisitsina NM , Forgacs E , Minna JD , Lisitsyn NA . LRP‐DIT, a putative endocytic receptor gene, is frequently inactivated in non‐small cell lung cancer cell lines. Cancer Res. 2000;60(7):1961‐1967. 10766186

[26] Tabouret E , Labussière M , Alentorn A , Schmitt Y , Marie Y , Sanson M . LRP1B deletion is associated with poor outcome for glioblastoma patients. J Neurol Sci. 2015;358(1–2):440‐443. 10.1016/j.jns.2015.09.345 26428308

[27] Langbein S , Szakacs O , Wilhelm M , et al. Alteration of the LRP1B gene region is associated with high grade of urothelial cancer. Lab Invest. 2002;82(5):639‐643. 10.1038/labinvest.3780458 12004004

[28] Prazeres H , Torres J , Rodrigues F , et al. Chromosomal, epigenetic and microRNA‐mediated inactivation of LRP1B, a modulator of the extracellular environment of thyroid cancer cells. Oncogene. 2011;30(11):1302‐1317. 10.1038/onc.2010.512 21057533

[29] Lu Y‐J , Wu C‐S , Li H‐P , et al. Aberrant methylation impairs low density lipoprotein receptor‐related protein 1B tumor suppressor function in gastric cancer. Genes Chromosom Cancer. 2010;49:412–424. 10.1002/gcc.20752 20095042

[30] Ni S , et al. Down expression of LRP1B promotes cell migration via RhoA/Cdc42 pathway and actin cytoskeleton remodeling in renal cell cancer. Cancer Sci. 2013;104(7):817‐825. 10.1111/cas.12157 23521319 PMC7657119

[31] Wang Z , Sun P , Gao C , et al. Down‐regulation of LRP1B in colon cancer promoted the growth and migration of cancer cells. Exp Cell Res. 2017;357(1):1‐8. 10.1016/j.yexcr.2017.04.010 28408316

[32] Nakagawa T , Pimkhaokham A , Suzuki E , Omura K , Inazawa J , Imoto I . Genetic or epigenetic silencing of low density lipoprotein receptor‐related protein 1B expression in oral squamous cell carcinoma. Cancer Sci. 2006;97(10):1070‐1074. 10.1111/j.1349-7006.2006.00283.x 16918994 PMC11159176

[33] Sonoda I , Imoto I , Inoue J , et al. Frequent silencing of *Low Density Lipoprotein Receptor‐Related Protein 1B (LRP1B*) expression by genetic and epigenetic mechanisms in esophageal squamous cell carcinoma. Cancer Res. 2004;64(11):3741‐3747. 10.1158/0008-5472.CAN-04-0172 15172977

[34] Kim EY , Lee JW , Yoo HM , Park CH , Song KY . The platelet‐to‐lymphocyte ratio versus neutrophil‐to‐lymphocyte ratio: which is better as a prognostic factor in gastric cancer? Ann Surg Oncol. 2015;22(13):4363‐4370. 10.1245/s10434-015-4518-z 25805235

[35] Ying H‐Q , Deng Q‐W , He B‐S , et al. The prognostic value of preoperative NLR, d‐NLR, PLR and LMR for predicting clinical outcome in surgical colorectal cancer patients. Med Oncol. 2014;31:305. 25355641 10.1007/s12032-014-0305-0

[36] Feng J‐F , Huang Y , Chen Q‐X . Preoperative platelet lymphocyte ratio (PLR) is superior to neutrophil lymphocyte ratio (NLR) as a predictive factor in patients with esophageal squamous cell carcinoma. World J Surg Onc. 2014;12(1):58. 10.1186/1477-7819-12-58 PMC3973187 24641770

[37] Diem S , Schmid S , Krapf M , et al. Neutrophil‐to‐Lymphocyte ratio (NLR) and Platelet‐to‐Lymphocyte ratio (PLR) as prognostic markers in patients with non‐small cell lung cancer (NSCLC) treated with nivolumab. Lung Cancer. 2017;111:176‐181. 10.1016/j.lungcan.2017.07.024 28838390

[38] Prabawa IPY , Bhargah A , Liwang F , et al. Pretreatment Neutrophil‐to‐Lymphocyte ratio (NLR) and Platelet‐to‐Lymphocyte Ratio (PLR) as a predictive value of hematological markers in cervical cancer. Asian Pac J Cancer Prev. 2019;20(3):863–868. 30912405 10.31557/APJCP.2019.20.3.863 PMC6825764

[39] Dirican A , Kucukzeybek BB , Alacacioglu A , et al. Do the derived neutrophil to lymphocyte ratio and the neutrophil to lymphocyte ratio predict prognosis in breast cancer? Int J Clin Oncol. 2015;20(1):70‐81. 10.1007/s10147-014-0672-8 24532163

